# A person-centred approach to further develop a digital tool (KOKU-Nut) by developing a nutrition game for older adults living in the community

**DOI:** 10.1186/s12877-025-06434-2

**Published:** 2025-11-18

**Authors:** Chloe French, Sorrel Burden, Yimin Tang, Emma Stanmore

**Affiliations:** 1 School of Health Sciences, Faculty of Biology, Medicine and Health, The University of Manchester, M13 9PL, Manchester, UK; 2 National Institute of Health and Care Research, Applied Research Collaboration - Greater Manchester (NIHR ARC-GM), Manchester, UK; 3 Manchester Academic Health Science Centre, Manchester, UK; 4 Manchester University NHS Foundation Trust, Manchester, UK; 5 Salford Care Organisation, Northern Care Alliance NHS Trust, Stott Lane, Salford, UK

**Keywords:** Older adults, Malnutrition, Digital health, Co-creation, Participatory design, Wellbeing

## Abstract

**Background:**

Digital tools embedded with behaviour change theories can encourage the successful implementation and maintenance of positive lifestyle changes. Keep on Keep up (KOKU) is a wellbeing app offering strength and balance exercises and educational games to raise awareness of fall prevention. We aimed to further develop KOKU using a person-centred approach to inform and improve the eating habits of older adults living in the community.

**Methods:**

Initially, adults aged 65 and over were recruited from assisted living facilities across Greater Manchester. Five focus groups involving 33 older adults (aged 69–96 years) were conducted between October and December 2022. A topic guide was used flexibly to understand factors that influence the groups eating habits and explore their thoughts around a digital tool to support nutritional intake. Conversations were audio-recorded, transcribed verbatim and analysed using an inductive thematic approach. Researchers then collaborated with designers to develop a nutrition game based on UK dietary guidelines and findings from the focus groups. The nutrition game was tested with older adults in January 2024 before making any necessary changes. The older adults provided feedback and completed the system usability scale (SUS).

**Results:**

Four themes emerged from the dataset. The main determinants of food intake were personal preference, perceptions of foods and changes as a result of age-related decline. The ability, engagement and willingness to use digital technology varied among participants but the majority had a positive attitude towards the digital platform. A matching pair’s card game was chosen, and the educational content was produced by the research team. A prototype of the nutrition game (KOKU-Nut) was tested with 10 older adults on an iPad provided by the research team. Feedback was generally positive, and the median SUS was 87.5 (IQR: 65, 95) out of 100 indicating the nutrition game had excellent usability.

**Conclusions:**

We anticipate this person-centred approach will improve the design of KOKU-Nut to empower and educate users to improve their diet. Future research should focus on maximising social inclusion and diversity.

**Supplementary Information:**

The online version contains supplementary material available at 10.1186/s12877-025-06434-2.

## Background

Lifespan is increasing and it is estimated that almost one fifth of the population in the United Kingdom (UK) are 65 years or older [1]. Nutrition is an important, modifiable and well-established risk factor that can mediate the ageing trajectory and increase the prevalence of chronic disease [2–4]. Improving dietary intake can also optimise health and quality of life increasing the number of disability-free life years [5].

It is estimated that 1.3 million people in the UK over the age of 65 suffer from malnutrition (undernutrition) with the majority (93%) living in the community [6]. The prevention of malnutrition is important given its prevalence and it can further contribute to physical decline, frailty, musculoskeletal conditions and immune senescence [7, 8]. The James Lind Alliance (JLA) methodology brings together those with personal or professional experience to identify and prioritise unanswered questions and inform the research agenda [9]. A JLA Priority Setting Partnership (JLA-PSP) was conducted in 2020 on the topic of malnutrition and screening with 250 contributors. The report identified that early intervention in vulnerable groups to prevent malnutrition was the number one research priority [10].

Digital technology is increasingly being encouraged to complement clinical approaches as it has the potential to make health systems more efficient and sustainable, translating into greater outreach and savings [11, 12]. Psychological literature indicates that while individual action and motivation is important, a framework that nudges individuals to make these changes should stem from behavioural and social science conceptual frameworks to encourage continued engagement and maximise impact. Behaviour change in older adults is likely to be challenging given that habits relating to food intake will have been established over the life course. Digital tools embedded with behaviour change theories can encourage the successful implementation and maintenance of positive lifestyle changes as they often consider social, emotional and cognitive factors [13–16]. A recent meta-analysis identified that apps can increase physical activity in older adults but very few randomised controlled trials (RCTs) have considered an app to support nutritional intake to prevent malnutrition in this population [17]. Keep on Keep up (KOKU) is a digital tool offering strength and balance exercises based on the Otago exercise programme [18] and the EAST (easy, attractive, social, timely) framework to support behaviour change [19]. Trials in the UK and US have found that KOKU has high usability with improved balance, health status and confidence (relating to falling) after six weeks of independent use [20–22]. Gamification shares similarities with established behaviour change techniques so can help with engagement with the digital platform and help promote positive health behaviours [16].

It is well established that diet, specifically protein intake can complement the effects of exercise in order to preserve and build muscle mass and strength [23, 24]. However, a recent scoping review identified a lack of digital tools to support nutritional status in relation to fall prevention among older adults [25]. Given this, we aimed to use the person-centred approach to develop an educational game to embed within KOKU to inform users (older adults living in the community) about malnutrition and healthy eating in line with UK dietary guidelines [26].

## Methods

The Consolidated Criteria for Reporting Qualitative Research (COREQ) guidelines were followed when designing, conducting and writing up this study (Supplementary material) [27].

### Study design

The digital platform was developed using a person-centred approach focusing on the views and feedback of potential end users [28, 29] by incorporating focus groups and usability testing. The development process included three phases focus groups with older adults; application development and initial application testing.

### Phase 1: focus groups with older adults

The first phase consisted of determining the product features and educational content required for the nutrition component of the digital intervention. A qualitative approach was undertaken such that older adults were invited to participate in a focus group. Focus groups were used to explore determinants of dietary intake through the experiences of the individuals [30]. To be included in a focus group, participants had to be at least 65 years old, live in the community and have the ability to give informed consent. Participants had to be able to communicate in English in order to understand the study information sheet, to provide informed consent and to participate in the discussion. Participants currently staying in a hospital or care home and those who had a known cognitive impairment were excluded.

#### Participant recruitment

We generated a sampling frame, and participants were recruited through purposive sampling, focusing on identifying a mix of male and female participants, with a range of ages (≥ 65 years) from across different districts of Greater Manchester. Participants were recruited from assisted living facilities as these typically house residents aged ≥ 55 years and offer self-contained homes (private or rented) within a larger complex. Individuals at these facilities are responsible for their own meal preparation and cooking however, there is often a communal lounge where residents are invited to coffee mornings and regular activities. To maximise variation in sampling [31], we recruited participants from assisted living facilities of different sizes and geographical areas (inner city and suburban). Recruitment of participants continued until data saturation was achieved [32]. Data collection and analysis happened simultaneously, so that discussions were compared against existing codes. This allowed data saturation to be determined such that subsequent discussions did not provide additional information or themes relating to the research question.

#### Data collection

Focus groups were conducted between October and December 2022 in the communal area of assisted living facilities. Each focus group consisted of six to eight older adults and lasted approximately 60 minutes in length. Focus groups were led by a female facilitator (CF) with a second researcher present (YT) to help moderate the discussion. The facilitator was a researcher with expertise in nutrition who had undertaken training in qualitative research and did not have a personal relationship with the participants. Before commencing the focus group, participants were reminded about the study’s aims and objectives, and the importance of confidentiality. Participants had the right to not participate in aspects of the discussion without providing an explanation and were reminded that they were welcome to take breaks as necessary. The discussion involved semi-structured questions, and the facilitator was guided by the focus group topic guide (Supplementary material), as well as questions that sought to clarify and elaborate on emerging themes. A research journal was kept (by CF) to add context, document thoughts and experiences and to allow for reflection on the focus groups. Each discussion was audio recorded and transcribed verbatim (by CF) removing all names and identifiable information prior to analysis. Audio recordings were destroyed after transcripts were verified. The transcript data was then uploaded and managed using NVivo Version 10 (QSR International Pty Ltd, Doncaster, VIC, Australia).

#### Ethical considerations

Ethical approval was obtained by The University of Manchester Research Ethics Committee on 22/07/22 (Ref: 2022-14724-24818) and complies with the Declaration of Helsinki. The study was conducted in accordance with the UK Policy Framework for Health and Social Care Research and other applicable guidance. All potential participants were provided with a detailed participant information sheet explaining the purpose of the study, their role as a participant, the potential risks and benefits of taking part and issues relating to confidentiality of data collection and reporting. Written informed consent was obtained from all participants prior to commencement in the research.

#### Data analysis

Transcript data was analysed thematically using an inductive approach [33, 34]. Transcripts were read repeatedly to identify and code themes found in the text. One researcher (CF) coded the data, and a second researcher (YT) independently reviewed 20% of the codes and an audit trail was maintained in NVivo to track coding iterations. Any discrepancies were solved through discussion with other researchers (SB, ES) to ensure consensus in interpretation and understanding. Similar codes were systematically grouped together to form a hierarchy of themes and subthemes. Subthemes captured variations within broader themes to allow the experiences, perspectives and context of different participants to be portrayed. A thematic map was used to provide an overview between and within themes to sufficiently capture the research question and overall dataset. Anonymised quotations provide examples of the data and highlight important points within each theme.

### Phase 2: application development

The second phase focused on the design and development of a nutrition game to educate and prompt older adults to improve their nutritional intake in line with UK dietary guidelines [26].

An agile design sprint was utilised as this methodology encourages collaboration, adaptability and innovation in a timely manner [35]. Agile design sprints provide context to the problem; generate a variety of ideas and consider potential solutions for the users based on the resources and time available [36]. Furthermore, design sprints have the potential to be cost effective as they involve gaining feedback on early versions of mocked up prototypes before resources are used to create full versions [35]. A collaborative workshop was held with researchers with expertise in digital interventions for older adults (ES) and nutrition (CF). Their role was to reflect (prioritise and share) ideas from phase one alongside nutrition guidelines and behaviour change theories. The research team worked with designers who have extensive experience in producing tools created alongside people with lived experiences. Throughout the workshop, a primary emphasis was placed on generating ideas for mini games that were evidence-based (educational), innovative and engaging. Individuals at the workshop ideated and shared ideas for new mini games tailored to raise awareness of the best evidence and subsequently nudge users to improve their diet. To facilitate the visualisation and communication of ideas, potential solutions (without the limitation of time, money or resources) were considered and captured on post it notes. Presentations were followed by constructive discussions, enabling feedback and refinement of the ideas through collaborative input and suggestions. A “Crazy 8s” agile design sprint task was then led by the design team [37] such that everyone at the workshop sketched eight different game concepts in eight minutes before each sharing their ideas with the group. This process helped to quickly generate a large number of ideas before considering potential features that would complement and improve the user experience. After the initial ideation workshop, a separate session was conducted with the design team who refined the preliminary ideas into four solidified games. These concepts were chosen as they can provide value to the user, whilst ensuring engaging gameplay and were feasible with the available resources.

### Phase 3: initial application testing

In order to assess the practical capabilities, features, and user experience, participants who had taken part in phase one were invited to participate in the initial application testing. The demo was tested on Testflight for iPad [38] on community-dwelling older adults with no restrictions on health conditions or experience of using digital technology. Individuals were presented with information about the need for the intervention and the current design (app). Participants were involved in reviewing the prototype and providing feedback both positive and negative on the game concept, ease of use and the educational content. The researcher took notes on any positive or negative feedback and any challenge points when navigating the digital platform. Ease of using KOKU-Nut was also assessed using the 10-item system usability scale (SUS) [39]. Each question has a response based on a Likert scale ranging from strongly agree to strongly disagree and the responses provide a score as a measure of the overall usability of the system. A SUS greater than 68 is considered above average, a SUS between 71 and 84 is considered good and a score ≥ 85 is considered excellent [40]. Participants received an honorarium in the form of a book voucher (£20.00 GBP).

## Results

### Focus group results (Phase 1)

Five focus groups each lasting approximately 60 minutes were conducted between October and December 2022. In total, 33 older adults (mean age 82.8 years, SD 8.3) attended a focus group, most participants were female (78.8%) and of White British ethnicity (93.9%). Demographic characteristics of study participants are presented in Table 1. Thematic analysis of the dataset resulted in four overarching core themes and a series of sub themes demonstrating the thoughts and opinions of the older adults (Supplementary material).

**Table 1:** Demographic characteristics of study participants (older adults living in the community)

	Frequency (%) (*N* = 33)
Mean (SD) Age	82.8 (8.3)
Gender: Male Female	7 (21.2) 26 (78.8)
Marital status: Single Separated/divorced Married Widowed	5 (15.2) 6 (18.2) 3 (9.1) 19 (57.6)
Ethnic origin: White British Black British	31 (93.9) 2 (6.1)

### Theme 1: change in diet due to age-related decline

This theme reflects how physiological ageing combined with psychosocial factors such as retirement and bereavement influence dietary behaviours. Many older adults noticed they had less of an appetite and this change in eating pattern was accompanied with an attitude of acceptance as an inevitable part of ageing.

*“I don’t know whether other people find this but I’ve found that as I’ve got older*,* I don’t get hungry” (P129*,* Female*,* aged 65–74)*

*So your diet does change to a certain extent as you’re getting older and that happens. (P134, male, aged 85+)*

It is well established that dietary habits are developed over the lifetime and often revolve around social cues. Older adults can often lose these cues, which can then alter intake. As well as these psychosocial factors, poor dentition was also identified as a physiological barrier and factor influencing food intake:

*“Once you start cooking for one*,* it’s never the same” (P137*,* Female*,* aged 85+)*.

*“When you’re alone*,* you just don’t have the appetite” (P136*,* Female*,* aged 85+)*.

*“Another thing that’s affected me in terms of eating*,* is my teeth*,* you know*,* they are no good… and there’s lots of them missing now” (P115*,* Female*,* aged 75–84)*.

Several participants were conscious of and sometimes restricted their fluid intake, especially in the evenings to prevent having to use the bathroom multiple times throughout the night. There was an awareness about the consequences of dehydration, but the decline and frustration associated with reduced bowel function was more of a concern and influencing factor when considering fluid intake amongst the sample.

*“I can’t drink after my evening meal… I’ve got a bladder problem*,* well a prostate problem actually which makes me want to go to the toilet every hour” (P134*,* Male*,* aged 85+)*.

*“It’s sort of a balancing act isn’t it*,* cause*,* you know*,* as you get older… you can be managing bladder problems” (P138*,* Male*,* aged 75–84)*.

Health complications were both prevalent and a concern for many participants and influenced dietary intake. Participants were aware and consistently mentioned foods they should avoid, however never focused on foods they should be eating to optimise their nutrition and health.

*“When I first became a diabetic*,* I did find it a bit difficult because I used to love an ice cream cake… and I had to stop all that” (P141*,* Male*,* aged 75–84)*.

*“I have to watch my cholesterol… because I have high blood pressure” (P116*,* Female*,* aged 85+)*.

### Theme 2: perception of foods

In general, there was a negative attitude towards pre-packaged and processed foods with a focus on the importance of home cooking:

*“I try to cook by myself*,* because then I know what’s going in it” (P122*,* Female*,* aged 65–74)*.

*“When you do it yourself*,* course it takes a lot longer*,* but I still prefer to do it that way” (P126*,* Female*,* aged 75–84)*.

Participants also had perceptions relating to particular food groups such as dairy and salt and their impact on health. This reflects nutrition messages previously promoted by the media rather than evidence-based advice targeted for this age-group.

*“I shouldn’t be having too much dairy but I’m afraid I do” (P111*,* Female*,* aged 75–84)*.

*“I’ve got to cut down on the salt” (P116*,* Female*,* aged 85+)*.

### Theme 3: food choice

Personal preference, ease of preparation and taste were also key determinants of food and fluid intake. This highlights the importance of personalisation for dietary interventions to encourage adherence amongst older populations. Personal preferences may be further heightened if the social environment changes and eating alone becomes more common leading to food choices being based more on ease and taste preference.

*“It is a mix of what I like but also how easy it is” (P129*,* Female*,* aged 65–74)*.

*“A lot of your diet does go on taste” (P134*,* Male*,* aged 85+)*.*“I find water boring*,* so I add blackcurrant juice” (P113*,* Male*,* aged 75–84)*.

*“Well I usually drink water*,* but it’s got to be absolutely cold out the fridge” (P121*,* Female*,* aged 75–84)*.

Older adults often experience declines in executive functioning, energy levels, dexterity and mobility which can influence food shopping and cooking ability. The following examples suggest that batch cooking was a popular technique used by this group.

*“Occasionally I’ll get a big pan of things and make a stew which will cover me for at least three days*,* maybe four if I stretch it out” (P138*,* Male*,* aged 75–84)*.

*“When I cook*,* I cook a certain amount and then freeze it so*,* then can take it out whenever I need to” (P116*,* Female*,* aged 85+)*.

### Theme 4: perspectives towards digital tools to support dietary intake

The final theme derived from the dataset demonstrates the nuance around technology use among older adults by exploring attitudes towards the digital intervention and possible features that could be incorporated into the nutritional component. Some people did not have access to iPads or smartphones and some people had access to but did not or could not use this technology.

*“I’ve got one that I need to use but I don’t know where to start with it” (P125*,* Female*,* aged 85+)*.

*“Though I must admit my patience with these things is getting worse and worse*,* what with all these bloody passwords and things” (P113*,* Male*,* aged 75–84)*.

Despite the initial resistance towards digital technology, many people were interested in learning with the appropriate support and guidance. The adoption of technology is likely to require adequate support and a clear value proposition such as access to personalised health advice or easier meal planning.

*“Well*,* I think I would like to learn because I’ve got to change my attitude to it and accept that it’s here to stay” (P138*,* Male*,* aged 75–84)*.

*“They’d have to be very patient and it would take a very long time*,* but yes I would like to learn” (P131*,* Female*,* aged 65–74)*.

The majority of participants had a positive attitude towards the digital health intervention with many offering questions or suggestions around what they should be eating and what they would like to see in the nutritional component. Suggestions fell into three main categories: education, recipes and motivation.

### Education

Many participants were engaged and interested in how they could improve their diet:

*“So where are you saying this proteins got to come from” (P138*,* Male*,* aged 75–84)*.

*“So what do I add to my porridge and toast?” (P134*,* Male*,* aged 85+)*.

*“I’m interested in why you should eat certain kinds of food… and what vitamins you need” (P129*,* Female*,* aged 65–74)*.

### Recipes

Many participants also highlighted that recipes would be a useful addition to the digital tool with some participants interested in simple recipes that required minimal cooking equipment.

*“Recipes*,* information*,* reminders*,* …like what veg to put in it*,* what’s easier to use or different veg’s that you can make in the same type of dish” (P138*,* Male*,* aged 75–84)*.

*“I wouldn’t mind one or two vegetarian recipes” (P136*,* Female*,* aged 85+)*.

*“We need more simple meals” (P124*,* Male*,* aged 65–74)*.

### Motivation

The dataset also highlighted the challenges around achieving behaviour change and identified that a motivational component within the digital tool would be beneficial to remind participants to stay on track.

*“It’s sticking to it; it’s the willpower that’s hard” (P127*,* Female*,* aged 85+)*.

*“It [notifications] would help me… just could say to you*,* have you drunk within the last whatever time or when was the last time you had a drink and then you can just say yay or nay… especially with your memory as you’re getting older” (P124*,* Male*,* aged 65–74)*.

### Result of application development (Phase 2)

Phase one demonstrated that poor dentition, lack of appetite and change in social environment can reduce the desire to eat within this population and increase the risk of malnutrition. These findings highlight that a digital tool tailored to support dietary intake and raise awareness of malnutrition among this population is a novel and important solution.

Four ideas were presented to the research team based on the ideation workshop. Following discussions between the digital agency and researchers (CF, ES), the matching pair’s card game was chosen. It was anticipated this would be the most engaging for the target audience, easy to understand and allows an educational component to be easily incorporated into the game mechanics to provide nutritional information and nudge older adults to choose certain foods. The educational content was produced by the research team based on lay language description of the current UK dietary guidelines (the Eatwell guide) [26], evidence from the literature [41, 42] and findings from Phase 1. Adherence to these guidelines remains low despite being associated with a range of health benefits including a reduced mortality risk [43, 44]. In particular, the app was designed to increase consumption of fruit and vegetables, lean protein and fibre whilst reducing intake of high fat and sugar foods. Phase one demonstrates a lack of awareness around the importance of consuming high-quality protein and there was negative messaging around the consumption of dairy foods which highlights a lack of age-specific and evidence-based nutrition messaging. The game is tailored to the requirements of older adults and includes simple tips if users are having difficulty chewing and practical advice for how to increase the protein content of commonly consumed foods such as toast. Information was also included about common signs of malnutrition and signposted on where to seek additional support.

**Fig. 1:**
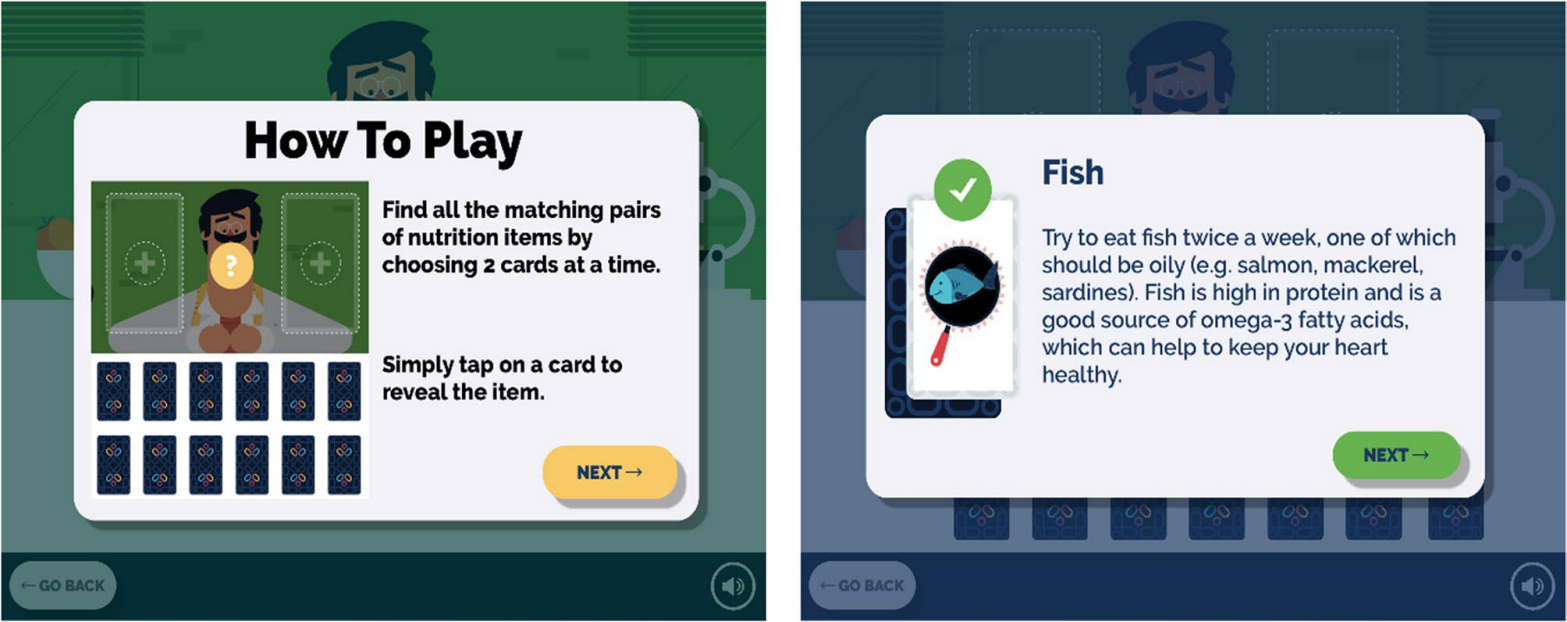
Screenshot of matching pair’s game available on KOKU-Nut

The graphics were developed by the design team using Apple Xcode using the Swift development language for iPad [45], and Android Studio using the Java development language for Android [46]. The game involves the user choosing a pair of cards from a possible twelve that are faced downwards, with the aim of finding all matching items (Fig. 1). The twelve cards when turned over show six different images of food or nutritional related content (with each food item appearing on two cards). If a matching pair is found, a pop up will appear presenting a larger image of the matching food item along with information about the item (Fig. 1). If the cards are a mismatch, a message appears to say “not a match” and the cards will return to their original face down position. The game is complete once all pairs have been matched, and the user is led to see important tips to improve diet and prevent malnutrition.

### Result of initial application testing (Phase 3)

A total of 10 end users tested the beta version of the app on an iPad provided by the research team. Testing took place face-to-face in January 2024 and involvement typically lasted 45 min. A think-aloud approach was utilised such that the researcher (CF) observed participants using the intervention who were encouraged to say their thoughts out loud in order to provide detailed insight into their interpretation, experiences and barriers of using the intervention. At the end of the testing period, users also completed the SUS to quantitatively evaluate the usability of the digital platform (KOKU-Nut) [39, 40]. The median SUS score was 87.5 (IQR: 65, 95) out of 100 indicating the intervention had excellent usability [40].

Feedback from end users about the game rather than issues from using the device was noted by the researcher (CF) and then discussed with the wider research team (ES, SB). Criteria for making modifications was based on if the feedback aligned with the evidence base, if the same issue was reported by multiple users or would be appropriate to a wider audience and if the changes could be easily incorporated into the game [47, 48]. Individuals generally had positive feedback, found the game enjoyable and the content clear, informative and interesting. Suggestions that were uncontroversial and easy to change such as the clarification of wording were implemented immediately. Other suggestions were more complex or nuanced and so the feedback was noted, and further testing will be conducted to guide future iterations. Table 2 highlights the feedback and amendments made to improve the clarity of the educational content and so that the instructions page was more intuitive for the user. This testing process allowed older adults to be active co-creators in the development of KOKU-Nut, highlighted issues that were not initially obvious to the research team and helped to ensure the final app was user friendly. Amendments confirmed by the research team were then incorporated by the design team and the game was developed for both Android and iOS to increase accessibility across a range of devices (iPads and tablets).

**Table 2:** Changes made to KOKU-Nut based on user feedback

Feedback from user	Action taken
Area for improvement	
One participant found the game too challenging and thought there were too many cards on the screen	Despite this comment other users thought the difficulty level was appropriate and were able to complete the game so the number of cards have not been changed
The word phytochemical is too technical	Phytochemicals has been replaced with vitamins
Not clear what 14 units of alcohol translates to	This has been removed
One participant suggested that a quiz at the end of the game would be helpful to test knowledge	This has been noted as a suggestion for a possible future iteration
A few participants missed the ‘next’ button on the instructions page and tried to play on this screen	Additional sentence has been added into instructions page to clearly direct users to start the game
One participant thought the game was very text heavy	Despite this comment, other users found the content informative and the right level so no changes were made
Thought the cards disappeared/faced down too quickly so it was difficult to see where cards went	If a matching pair is not found, cards now return to face down position one by one rather than at the same time
A few participants found the term sarcopenia too technical	The term sarcopenia has been removed so now just says “to help maintain muscle mass and strength”
One participant thought that meat should also be included as an example of processed food.	This has been updated to include bacon as an example of an ultra-processed food
Positive feedback	
Found the game difficult at first but then really enjoyed it “would rather do this than watch the soaps”	-
Liked information about canned/frozen fruit and vegetables	-
Thought it was very informative, entertaining and not patronising	-
“I love it and would recommend it to my friends”	-
“The advice contained in KOKU would be highly beneficial to the user… I am convinced it would enhance the lives of many people”	-
“KOKU is both stimulating and enjoyable. It brings great satisfaction and most importantly promotes good health”	-

## Discussion

This study has demonstrated the process of using a person-centred approach to create a digital nutrition game with and for older adults living in the community with input from a multi-disciplinary team of experts. A qualitative approach was utilised in phase one as recommended by the literature [49, 50] to explore the current attitudes and determinants of dietary habits in older adults, to provide context to the intervention and to identify how to manipulate future food choices to improve intake. These findings were then used to inform phase two (the development of KOKU-Nut) by prioritising and incorporating user perspectives wherever possible alongside best evidence to support behaviour change [16, 51] to align with nutritional guidelines [26]. Application testing in phase three allowed changes to be incorporated into the digital platform to support the preferences of end users (community-dwelling older adults).

This person-centred approach has been shown to improve the relevance and overall quality of the research to ensure that it prioritises and considers aspects that are important to the people the research is aimed at [52, 53]. We anticipate this consideration at all stages of the design process has contributed to the high usability score and will support acceptability and use of the digital platform by older adults. Moreover, digital tools have the potential to further marginalise and exclude vulnerable members of the population including groups of older adults. The person-centred approach involves users in the design process and helps to mitigate barriers regarding accessibility to technology and the user interface.

Phase one identified that the main determinants of intake were change in diet due to age-related decline (psychosocial and physiological factors such as change to living situation and poor oral health), their perception of foods (food quality, health benefits of foods) and food choice (taste, cooking habits). These findings are consistent with the wider literature and reflect the complexity of food intake demonstrating the influence from physical, psychological and social change that occurs in old age [54, 55]. Participants in phase one were aware of the importance of food on health status, but not how intake should change with age. It became apparent that dietary advice received by this population throughout their adult life was still a concern. This highlights the rationale for developing a specialist and evidence-based tool targeted for older adults given the additional nutritional challenges within this population [42]. Participants also spoke about having a reduced appetite, often termed anorexia of ageing and this is likely to contribute to the risk of malnutrition [56]. Malnutrition is a challenging health concern that commonly occurs in older adults [6, 57, 58] but awareness among this population group remains low [59]. Findings from phase one informed the educational component within KOKU-Nut to provide messaging around areas such as unplanned weight loss and guidelines for dairy consumption.

In phase one, participants’ also highlighted barriers to using technology including a lack of knowledge around these devices, low confidence and the excessive use of passwords. Despite some concerns around the use of digital technology, recent evidence has shown that age does not appear to be a barrier for use [60]. A review by the Organization for the Review of Care and Health Apps (ORCHA), a digital health evaluation and distribution organization found that more than half (52%) of people surveyed who were aged 65 years and older support the move to digital health [61]. Digital tools are likely to help engage hard to reach and vulnerable groups who may struggle or feel excluded from traditional face-to-face approaches [61]. Moreover, the development of the nutrition game was guided by the thoughts, needs and ideals of potential end users. This form of participatory design [62] helps to maximise the acceptability, usability and effectiveness of an intervention amongst users and is particularly important when considering the specific requirements of older adults [16, 63, 64]. A review of studies including older people in the design process of technology innovations showed that user involvement can prevent ageism, create a positive sense of ownership and improve the quality of the intervention [65].

Findings from phase three demonstrated that potential end users liked the concept, information and graphics and reported the nutrition game to have high usability. A systematic review including 46 studies reported that gamification makes a typically boring activity more enjoyable, competitive and engaging [66]. Gamification can be defined as entertaining the user as they learn or change behaviour [67]. This approach may help to promote and encourage healthy behaviours through goal setting, feedback on performance, progress tracking and problem-solving, which share key elements with established behaviour change techniques [16, 68]. The educational content provides knowledge to the user (community-dwelling older adults) about how and why they should be eating to support healthy ageing which increases the rationale and motivation for changing their behaviour. Furthermore, the problem-solving and gamification may contribute to increased engagement and the enjoyment experienced by users may encourage more regular use of the digital platform.

The combined effect of resistance exercise with daily protein supplementation has been shown to improve muscle mass, strength, quality of life and body composition [69, 70]. Future research should consider if this digital platform comprising strength and balance exercises along with nutrition information can improve health indicators and quality of life.

### Strengths and limitations

There were several limitations to this study including that it was a relatively homogeneous sample, the majority of which were white British females. This ethnic profile reflects the population of older adults living in England and Wales in 2021 [71] however the socio-cultural influences on eating behaviour would be better assessed by having a more targeted recruitment technique to obtain a culturally diverse group from different socio-economic backgrounds. We have been working on methods to further engage underserved populations such as South Asian communities [72] and will continue to work to overcome these barriers in future research to mitigate under representation of ethnic minority groups and increase the generalisability of findings.

Another limitation is that important characteristics of participants were not collected including educational level, anthropometry, risk of malnutrition and digital literacy. Furthermore, it is likely that qualitative findings were influenced by social desirability bias, given that nutritional information is often misreported [73] and participants in the current study were aware that they were speaking to a nutritionist. Objective measures such as dietary surveillance can improve the precision of data and provide a more accurate measure of intake; however, this involves large administrative costs, participant burden and intrusion. It would have been beneficial to consider and explore other stakeholders’ perspectives (such as dietitians, geriatric consultants, community outreach teams and family members or friends) as part of the design process; however, this was not possible due to time and financial limitations. The SUS included as part of the usability testing in phase three does not assess motivation or the user experience over time. Therefore, the ability to assess long-term engagement or potential for behaviour change is limited. Moreover, the data collection and analysis components were subject to observer bias given that authors conducted multiple parts of this study. It would be beneficial for future work to incorporate additional researchers to act as trained moderators separate from the coders to minimise the observer-expectancy effect and improve interrater reliability.

The main strength of this study was the engagement of participants at both the needs assessment and initial application testing phase. This interaction and feedback allowed the user’s needs, desires and abilities to be considered and incorporated into the design process. In addition, this is a novel and innovative digital platform that incorporates gamification which is likely to increase enjoyment and engagement.

## Conclusion

We anticipate this person-centred design approach will have improved the design of KOKU-Nut. We plan to continue to develop and iteratively revise the app to maximise its potential and usefulness. Future research should seek to study more ethnically diverse perspectives given the influence of culture and upbringing on dietary habits and the heterogeneity of the sample in the current study.

## Supplementary Information

Supplementary material 1.

## Data Availability

The data presented in this study are available on request from the corresponding author.

## Abbreviations

UK: United kingdom
JLA: PSP-James lind alliance priority setting partnership
KOKU: Keep on keep up
COREQ: Consolidated criteria for reporting qualitative research
KOKU: Nut-keep on keep up nutrition
SUS: System usability scale
ORCHA: Organization for the review of care and health apps
