# Supplementary material for: A person-centred approach to further develop a digital tool (KOKU-Nut) by developing a nutrition game for older adults living in the community
Source: BMC Geriatr. 2025 Nov 18;25:925. doi: 10.1186/s12877-025-06434-2 (PMC12625430; doi:10.1186/s12877-025-06434-2)
Supplement: Supplementary file 1 — Supplementary material 1. [file 12877_2025_6434_MOESM1_ESM.docx]

**Supplementary information**

**Supplementary material 1-** COREQ checklist

| **Domain 1: Research team and reflexivity** |  | Location in manuscript (Section - page no.) |
| --- | --- | --- |
| **Personal Characteristics** |  |  |
| 1. Interviewer/ facilitator. Which author conducted the interview of focus group? | CF | Methods – 4 |
| 1. Credentials. What were the researcher’s credentials? | MSc MA (Hons) ANutr (PhD student) | - |
| 1. Occupation. What was their occupation at the time of the study? | PhD student | - |
| 1. Gender. Was the researcher male or female? | Female | Methods- 4 |
| 1. Experience and training. What experience or training did the researcher have? | Expertise in nutrition and training in qualitative research | Methods- 4 |
| **Relationship with participants** |  |  |
| 1. Relationship established. Was a relationship established prior to study commencement | No, the facilitator did not have a personal relationship with any of the participants | Methods - 4 |
| 1. Participant knowledge of the interviewer. What did the participants know about the researcher? E.g. personal goals, reasons for doing the research | Participants were briefed on the purpose of the study and understood that it was a research project being undertaken as part of a PhD project. Participants reviewed the participant information documentation prior to giving their written informed consent to be involved. | - |
| 1. Interviewer characteristics. What characteristics were reported about the interviewer/ facilitator? E.g. Bias, assumptions, reasons and interests in the research topic | See above (7) | - |
| **Domain 2: Study design** |  |  |
| **Theoretical framework** |  |  |
| 1. Methodological orientation and Theory. What methodological orientation was stated to underpin the study? E.g. grounded theory, discourse analysis, ethnography, phenomenology, content analysis | Underpinned by phenomenology | Methods- 3 |
| **Participant selection** |  |  |
| 1. Sampling. How were the participants selected? E.g. purposive, convenience, consecutive, snowball | Purposive sampling | Methods- 3 |
| 1. Method of approach. How were participants approached? E.g. face-to-face, telephone, mail, email | Face-to-face | Methods- 3 |
| 1. Sample size. How many participants were in the study? | 33 | Results - 6 |
| 1. Non-participation. How many people refused to participate or dropped out? Reasons? | Of the participants who agreed to participate and provided consent none refused to participate, withdrew consent or dropped out. | - |
| **Setting** |  |  |
| 1. Setting of data collection. Where was the data collected? E.g. home, clinic, workplace | Data was collected in a communal space at each of the assisted living facilities | Methods - 4 |
| 1. Presence of non-participants. Was anyone else present besides the participants and researchers? | Yes, a second researcher was present to help moderate discussion | Methods- 4 |
| 1. Description of sample. What are the important characteristics of the sample? E.g. demographic data, date | Mean age 82.8 years (SD 8.3 years). 26 females and 7 males. | Results - 6  Table 1 |
| **Data collection** |  |  |
| 1. Interview guide. Were questions, prompts, guides provided by the authors? Was it pilot tested? | Facilitator was guided by focus group topic guide | Supplementary information |
| 1. Repeat interviews. Were repeat interviews carried out? If yes, how many? | No | - |
| 1. Audio/ visual recording. Did the research use audio or visual recording to collect the data? | The focus groups were audio-recorded using an encrypted Dictaphone. | Methods -4 |
| 1. Field notes. Were field notes made during and/ or after the interview or focus group? | A research journal was kept | Methods - 4 |
| 1. Duration. What was the duration of the interviews or focus groups? | Focus groups ranged from 27:46 to 68:34 (minutes: seconds) | Results - 6 |
| 1. Data saturation. Was data saturation discussed | Recruitment of participants continued until data saturation was achieved i.e. when discussions did not provide additional information or themes relating to the research question | Methods - 4 |
| 1. Transcripts returned. Were transcripts returned to participants for comment and/ or correction? | No | - |
| **Domain 3: analysis and findings** |  |  |
| **Data analysis** |  |  |
| 1. Number of data coders. How many data coders coded the data? | One coded the data, second researcher reviewed 20% | Methods- 5 |
| 1. Description of the coding tree. Did authors provide a description of the coding tree? | No | - |
| 1. Derivation of themes. Were themes identified in advance or derived from the data? | Themes were derived from the data using an inductive approach | Methods - 5 |
| 1. Software. What software, if applicable, was used to manage the data | NVivo version 10 | Methods- 4 |
| 1. Participant checking. Did participants provided feedback on the findings? | No | - |
| **Reporting** |  |  |
| 1. Quotations presented. Were participant quotations presented to illustrate the themes/ findings? Was each quotation identified? E.g. participant number | Yes, specific comments were supported with direct quotes attributed to anonymised participant by gender, age and focus group | Results – 7 to 12 |
| 1. Data and findings consistent. Was there consistency between the data presented and the findings? | Yes, findings were consistent with wider literature | Discussion - 14 |
| 1. Clarity of major themes. Were major themes clearly presented in the findings? | Yes | Table 2 |
| 1. Clarity of minor themes. Is there a description of diverse cases or discussion of minor themes? | Yes, sub-themes are discussed in the manuscript | Table 2 |

**Supplementary material 2-** Focus group topic guide

Welcome/ briefing and initial questions: (10 minutes)

- Introduce myself
- Thank participants for getting involved
- Aim of the focus group: understand interest in mhealth, current barriers and what features would like to be included in digital tool / in what format
- Ensure information sheet has been read and take consent
- Collect demographic information from participants (See invitation letter for data being collected). The invitation letter will have been completed by participants in advance and will be collected at the start of the focus group.
- Ensure confidentiality (amongst each other and from a researcher perspective) and highlight option to stop at any time

Discussion 1 (15 minutes):

- Ask if people use digital technology or mhealth apps?
  - If yes, what do you use/ for what/ what do you like/ dislike
  - If no, what are barriers to using technology? Why don’t use?
  - Would you use an app to improve health?

Discussion 2 (30 minutes):

- How would you describe healthy ageing?
- Do you have any health and fitness goals?
  - If yes, what are they?
  - If no, why not?
- Perception of eating healthy
  - Do you think about what you eat?
  - Know the importance of eating healthy and drinking sufficient amounts
  - Do you think you have a healthy diet/ conscious of your diet or weight?
  - Barriers to eating healthy (preparation of food, enjoyment of meal times, lack of appetite)
  - How many meals/ drinks do you have a day? What is a typical day of eating
  - Thoughts on cooking meals from scratch/ microwave meals/ meals on wheels

Discussion 3 (15 minutes)

- If you could develop an app to improve nutritional status, what would you like to include? Why- what would be useful
- What would they like/ dislike
- Would they use it/ what aspect most important to them

Summary (5-10 minutes):

- Ask if people happy to be contacted in the future to assess updates/ future designs
- Explain how you will analyse and share the data
- Thank participants for their time and opinions

NB: malnutrition not always understood- use ‘eating less’ ‘unplanned weight loss’ and ‘poor appetite’

**Supplementary material 3**- Summary of themes, sub-themes, codes and example quotes

| **Overarching theme** | **Sub-theme** | **Codes** | **Example quotes** |
| --- | --- | --- | --- |
| Change in diet due to age-related decline | Physiological factors | - Being old - Appetite - Assistance - Lack of mobility - Health condition - Taste - Fluid - Meat | *“I don’t know whether other people find this but I’ve found that as I’ve got older, I don’t get hungry” (P129, Female, aged 65-74)*  *“So your diet does change to a certain extent as you’re getting older and that happens”*  *(P134, Male, aged 85+)*  *“I can’t drink after my evening meal… I’ve got a bladder problem, well a prostate problem actually which makes me want to go to the toilet every hour” (P134, Male, aged 85+)*  *“It’s sort of a balancing act isn’t it, cause, you know, as you get older… you can be managing bladder problems” (P138, Male, aged 75-84)*  *“When I first became a diabetic I did find it a bit difficult because I used to love an ice cream cake… and I had to stop all that” (P141, Male, aged 75-84)*  *“I have to watch my cholesterol… because I have high blood pressure” (P116, Female, aged 85+)* |
|  | Social factors | - Cooking - Community - Being old - Assistance - Emotional/ social | *“Once you start cooking for one, it’s never the same” (P137, Female, aged 85+)*  *“When you’re alone, you just don’t have the appetite” (P136, Female, aged 85+)*  *“Another thing that’s affected me in terms of eating, is my teeth, you know, they are no good… and there’s lots of them missing now” (P115, Female, aged 75-84)* |
| Perception of foods | Shop bought vs homemade | - Cooking - Food variety - Food quality - Cost | *“I try to cook by myself, because then I know what’s going in it” (P122, Female, aged 65-74)*  *“When you do it yourself, course it takes a lot longer but I still prefer to do it that way” (P126, Female, aged 75-84)* |
|  | Health claims | - Protein - Weight - Salt - Dairy | *“I shouldn’t be having too much dairy but I’m afraid I do” (P111, Female, aged 75-84)*  *“I’ve got to cut down on the salt” (P116, Female, aged 85+)* |
| Food choice | Personal preference | - Meat - Vegetarian - Food variety - Food quality - Portion sizes - Sweet food - Fruit and vegetables - Food availability/ ease - Taste | *“It is a mix of what I like but also how easy it is” (P129, Female, aged 65-74)*  *“A lot of your diet does go on taste” (P134, Male, aged 85+)*  *“I find water boring, so I add blackcurrant juice” (P113, Male, aged 75-84)*  *“Well I usually drink water, but it’s got to be absolutely cold out the fridge” (P121, Female, aged 75-84)* |
|  | Cooking habits | - Assistance - Lack of mobility - Food variety - Cost - Cooking | “*Occasionally I’ll get a big pan of things and make a stew which will cover me for at least three days, maybe four if I stretch it out” (P138, Male, aged 75-84)*  *“When I cook, I cook a certain amount and then freeze it so, then can take it out whenever I need to” (P116, Female, aged 85+)* |
| Perspectives towards digital tool to support nutritional intake | Attitude towards technology | - Fear of technology - Interest to use technology - Inability to use technology - Use of technology | *“I’ve got one that I need to use but I don’t know where to start with it” (P125, Female, aged 85+)*  *“Though I must admit my patience with these things is getting worse and worse, what with all these bloody passwords and things” (P113, Male, aged 75-84)*  *“Well I think I would like to learn because I’ve got to change my attitude to it and accept that it’s here to stay” (P138, Male, aged 75-84)*  *“They’d have to be very patient and it would take a very long time, but yes I would like to learn” (P131, Female, aged 65-74)* |
|  | Advance nutritional knowledge | - Supplements - Protein - Nutrition query | *“So where are you saying this proteins got to come from” (P138, Male, aged 75-84)*  *“So what do I add to my porridge and toast?” (P134, Male, aged 85+)*  *“I’m interested in why you should eat certain kinds of food… and what vitamins you need” (P129, Female, aged 65-74)* |
|  | Recipes | - Cooking - Health condition - Food quality | *“Recipes, information, reminders, …like what veg to put in it, what’s easier to use or different veg’s that you can make in the same type of dish” (P138, Male, aged 75-84)*  *“I wouldn’t mind one or two vegetarian recipes” (P136, Female, aged 85+)*  *“We need more simple meals” (P124, Male, aged 65-74)* |
|  | Increase motivation | - Being old - Health condition - Cooking | *“It’s sticking to it; it’s the willpower that’s hard” (P127, Female, aged 85+)*  *“It [notifications] would help me... just could say to you, have you drunk within the last whatever time or when was the last time you had a drink and then you can just say yay or nay... especially with your memory as you’re getting older” (P124, Male, aged 65-74)* |
